# Establishment and Characterization of Multi-Drug Resistant p53-Negative Osteosarcoma SaOS-2 Subline

**DOI:** 10.3390/diagnostics13162646

**Published:** 2023-08-11

**Authors:** Sergei Boichuk, Firyuza Bikinieva, Elena Valeeva, Pavel Dunaev, Maria Vasileva, Pavel Kopnin, Ekaterina Mikheeva, Tatyana Ivoilova, Ilshat Mustafin, Aigul Galembikova

**Affiliations:** 1 Department of Pathology, Kazan State Medical University, Kazan 420012, Russia; firuza1995@mail.ru (F.B.); dunaevpavel@mail.ru (P.D.); miheeva.1973@bk.ru (E.M.); 89179027099@mail.ru (T.I.); ailuk000@mail.ru (A.G.); 2 ”Biomarker” Research Laboratory, Institute of Fundamental Medicine and Biology, Kazan Federal University, Kazan 420008, Russia; 3 Department of Radiotherapy and Radiology, Russian Medical Academy of Continuous Professional Education, Moscow 125993, Russia; 4 Central Research Laboratory, Kazan State Medical University, Kazan 420012, Russia; elena.valeeva@kazangmu.ru; 5 Cytogenetics Laboratory, Carcinogenesis Institute, N.N. Blokhin National Medical Research Center of Oncology, Moscow 115478, Russia; mvnovikova94@mail.ru (M.V.); pbkopnin@mail.ru (P.K.); 6 Department of Biochemistry, Kazan State Medical University, Kazan 420012, Russia; ilshat.mustafin@kazangmu.ru

**Keywords:** Osteosarcoma (OS), ABC-transporters, paclitaxel, doxorubicin, resistance, apoptosis, DNA damage response (DDR)

## Abstract

Aim: To establish a p53-negative osteosarcoma (OS) SaOS-2 cellular subline exhibiting resistance to specific chemotherapeutic agents, including topoisomerase II inhibitors, taxanes, and vinca alkaloids. Methods: The OS subline exhibiting resistance to the chemotherapeutic agents indicated above was generated by the stepwise treatment of the parental SaOS-2 cell line with increasing concentrations of doxorubicin (Dox) for 5 months. Half-inhibitory concentrations (IC_50_) for Dox, vinblastine (Vin), and paclitaxel (PTX) were calculated by a colorimetric MTS-based assay. Crystal violet staining was used to assess cellular viability, whereas the proliferation capacities of cancer cells were monitored in real-time by the i-Celligence system. Expression of apoptotic markers (e.g., cleaved PARP and caspase-3), DNA repair proteins (e.g., ATM, DNA-PK, Nbs1, Rad51, MSH2, etc.), and certain ABC transporters (P-glycoprotein, MRP1, ABCG2, etc.) was assessed by western blotting and real-time PCR. Flow cytometry was used to examine the fluorescence intensity of Dox and ABC-transporter substrates (e.g., Calcein AM and CMFDA) and to assess their excretion to define the activity of specific ABC-transporters. To confirm OS resistance to Dox in vivo, xenograft experiments were performed. Results: An OS subline generated by a stepwise treatment of the parental SaOS-2 cell line with increasing concentrations of Dox resulted in an increase in the IC_50_ for Dox, Vin, and PTX (~6-, 4-, and 30-fold, respectively). The acquisition of chemoresistance in vitro was also evidenced by the lack of apoptotic markers (e.g., cleaved PARP and caspase-3) in resistant OS cells treated with the chemotherapeutic agents indicated above. The development of the multidrug resistance (MDR) phenotype in this OS subline was due to the overexpression of ABCB1 (i.e., P-glycoprotein) and ABCC1 (i.e., multidrug resistance protein-1, MRP-1), which was evidenced on both mRNA and protein levels. Due to increased expression of MDR-related proteins, resistant OS exhibited an excessive efflux of Dox. Moreover, decreased accumulation of calcein AM, a well-known fluorescent substrate for both ABCB1 and ABCC1, was observed for resistant OS cells compared to their parental SaOS-2 cell line. Importantly, tariquidar and cyclosporin, well-known ABC inhibitors, retained the intensity of Dox-induced fluorescence in resistant SAOS-2 cells. Furthermore, in addition to the increased efflux of the chemotherapeutic agents from Dox-resistant OS cells, we found higher expression of several DNA repair proteins (e.g., Rad51 recombinase, Mre11, and Nbs1, activated forms of ATM, DNA-PK, Chk1, and Chk2, etc.), contributing to the chemoresistance due to the excessive DNA repair. Lastly, the in vivo study indicated that Dox has no impact on the SaOS-2 Dox-R xenograft tumor growth in a nude mouse model. Conclusions: An acquired resistance of OS to the chemotherapeutic agents might be due to the several mechanisms undergoing simultaneously on the single-cell level. This reveals the complexity of the mechanisms involved in the secondary resistance of OS to chemotherapies.

## 1. Introduction

Osteosarcoma (OS) is a well-known aggressive malignant bone tumor that occurs frequently in children and adolescents. Combined therapy (surgery with pre- and postoperative chemotherapy) of localized (non-metastatic) OS significantly improved the patient outcomes [1], whereas disease-free survival and overall survival rates for the patients with advanced and recurrent OS remain unfavorable, in particular due to the rapid development of the multidrug resistance (MDR) of OS to the chemotherapeutic agents [2] and the lack of targeted therapies introduced into the clinical practice for OS patients. In general, the mechanisms of acquired resistance to chemotherapies include a broad spectrum of universal and overlapping mechanisms rendering the tumors non- or less susceptible to a broad spectrum of anti-cancer agents. This includes overexpression of drug efflux pumps in cancer cells [3,4], reduced uptake of the drug, enhanced DNA damage response (DDR) [5], deregulation of apoptosis [6,7,8], epithelial-mesenchymal transition [9,10,11], etc. Of note, these mechanisms can also overlap, especially at the final stage of the disease.

p53 is a tumor suppressor gene that is mutated in ~20% of OS and frequently undergoes rearrangement, resulting in the loss of its expression. The most common mechanisms of p53 inactivation in OS include rearrangements and MDM2 mutations and amplifications [12,13,14]. In general, OS patients with TP53 mutations had poorer overall survival [15]. Whereas the effectiveness of cancer chemotherapy correlates with the ability to induce a p53-dependent apoptotic response [16], it is still debatable whether overexpression of wild-type p53 modulates chemosensitivity in MDR OS cells [17]. 

The aim of the present study was to examine the mechanisms involved in the acquired resistance of p53-negative OS to certain chemotherapeutic agents, including doxorubicin (Dox) and paclitaxel (PTX). To achieve this goal, we established the Dox-resistant SaoS-2 subline (further named SaOS-2_DoxR) from parental OS cells. The resistance of SaOS-2_DoxR to certain chemotherapeutic drugs, including Dox, PTX, and vinblastine (Vin), was due to the increased expression and activity of ABC-transporters, in particular P-glycoprotein, thereby facilitating an efflux of Dox from cancer cells. In addition to the upregulation of ABC-transporters, SaOS-2_DoxR cells exhibited the signatures of activated DDR-signaling pathways involved in the repair of double-strand breaks (DSBs). This was shown for both homology-mediated DNA repair and non-homologous end-joining (NHEJ) pathways.

Thus, our established and characterized SaOS-2_DoxR cell subline exhibiting cross-resistance to certain chemotherapeutic agents via pleiotropic molecular mechanisms can be a useful tool for identifying novel mechanisms of drug resistance and new drug targets in OS.

## 2. Methods

### 2.1. Chemical Compounds

PTX, Dox, Vin, and Ifosfamide (Ifos) were obtained from Sigma-Aldrich (Merck KGaA, Darmstadt, Germany). Calcein acetoxymethyl ester (Calcein AM) and Green CMFDA were obtained from Abcam (Abcam, Cambridge, MA, USA). Tariquidar, Cyclosporin, and MK-571 were purchased at SelleckChem (Houston, TX, USA). The chemicals were dissolved in dimethyl sulfoxide (DMSO) according to the manufacturer’s recommendations. For in vivo experiments, the stock solution of Dox (1 mg/mL) was prepared in DMSO and diluted before administration in a formulation that contained a final concentration of 5% DMSO, 40% PEG-400, 5% Tween 80 (Sigma-Aldrich, Merck KGaA, Darmstadt, Germany), and 50% dd H_2_O. 

### 2.2. Cell Lines and Culture Conditions

The OS cell line SaOS-2 was obtained from the American Type Culture Collection (Manassas, VA, USA). The Dox-resistant OS subline (SaOS-2_DoxR) was established via the stepwise treatment of OS cells with increasing concentrations of Dox (using a maximum dose of 1 μM). Both OS cell lines were maintained in RPMI-1640 medium (Paneco, Moscow, Russia) supplemented with 15% fetal bovine serum (Gibco; Thermo Fisher Scientific, Inc., Waltham, MA, USA), 50 U/mL penicillin, and 50 μg/mL streptomycin (Paneco, Moscow, Russia). The cells were cultured at 37 °C in a humidified atmosphere of 5% CO_2_ in an incubator (LamSystems, Miass, Russia). 

### 2.3. Antibodies

The primary antibodies used for western blotting were anti-PARP (cat. no. 436400; Invitrogen; Thermo Fisher Scientific, Inc., Waltham, MA, USA), cleaved caspase-3 (cat. no. 9661S), p-BRCA1 S1524 (cat. no. 9009S), MSH2 (cat. no. 2017T), MSH6 (cat. no. 5424), Chk1 (cat. no. 2360S), Chk2 (cat. no. 6334P), Topoisomerase IIα (cat. no. 12286T), Rad51 (cat. no. 8875S), ERCC1 (cat. no. 12345), XRCC1 (cat. no. 2735), (Cell Signaling Technology Inc., Danvers, MA, USA); anti-ABC subfamily G member 2 (cat. no. sc-58222), MDR-1 (cat. no. sc-55510), MRP-1 (cat. no. sc-18835), BRCA1 (cat. no. sc-642) (Santa Cruz Biotechnology, Dallas, TX, USA), and beta-actin (cat. No. A00730-200, GenScript, Piscataway, NJ, USA); p-ATM S1981 (cat. no. ab81292), ATM (cat. no. ab32420), p-DNA-PK S2056 (cat. no. ab18192), DNA-PK (cat. no. ab32566), MGMT (cat. no. ab108630), (Abcam plc., Cambridge, UK); pChk1 S317 (cat. no. AF2054), pChk2 T68 (cat. no.AF1626), (R&D Systems, Inc., R&D Systems, Inc. Minneapolis, MN, USA), Nbs1 (cat. no. NB100-143), Mre11 (cat. no. NB100-141), (Novus Biologicals, Centennial, CO, USA). HRP-conjugated secondary antibodies, anti-mouse immunoglobulin (Ig)G (cat. no. sc-2005) and anti-rabbit IgG (cat. no. sc-2004), were purchased from Santa Cruz Biotechnology, Dallas, TX, USA. 

### 2.4. Western Blotting Analysis

To examine the protein expression in parental and Dox-R cells, whole-cell lysates (WCL) were prepared by scraping the cells growing as monolayers into radio-immunoprecipitation buffer (RIPA buffer) (25mMTris-HCl pH 7.6, 5mMnEDTA, 150mMNaCl, 0.1% SDS, 1% NP-40, 1% sodium deoxycholate) supplemented with a cocktail of protease and phosphatase inhibitors. The cellular lysates were further incubated for 1 h at 4 °C and clarified by centrifugation for 30 min at 11,400 rpm at 2 °C. The protein concentrations in WCL were calculated by the Bradford assay. The protein samples (20 µg) were loaded on the 4–12% Bis-Tris or 3–8% Tris-acetate NuPAGE gels (Invitrogen, Carlsbad, CA, USA) and, upon completion of electrophoresis, transferred to a nitrocellulose membrane (Bio-Rad, Hercules, CA, USA). Membranes were probed with primary (1:1000 and incubated overnight at 4 °C) and secondary antibodies (1:1000 and incubated for 1 h at room temperature) and visualized by enhanced chemiluminescence (Western Lightning Plus-ECL reagent, Perkin Elmer, Waltham, MA, USA). The densitometry analysis of Western blotting images was performed by NIH ImageJ software, version 1.49 (Bethesda, MD, USA).

### 2.5. Crystal Violet Staining

Cells were seeded into the culture plates and treated with PTX (1 µM), Vin (0.1 µM), and Dox (0.5 µM) for 96 hrs. Cell medium was aspirated, and 5 mL of 0.5% crystal violet staining solution was added to each well and incubated for 20 min at room temperature. Plates were washed four times in a stream of tap water and kept inverted on filter paper to air-dry 3 mL of 1% SDS was added to each plate and kept on a shaker at room temperature for 1 h. The optical density was measured at 570 nm wavelength using a MultiScan FC plate reader (Thermo Fisher Scientific, Waltham, MA, USA).

### 2.6. RNA Extraction and Reverse Transcription-Quantitative Polymerase Chain Reaction (RT-qPCR)

Parental and SaOS-2_DoxR cells were seeded in p100 plates and cultured for 48 h. TRIzol reagent (cat. no. BC032; Invitrogen; Thermo Fisher Scientific, Inc.) was used to extract total RNA, according to the manufacturer’s protocol, and re-suspended in diethyl pyrocarbonate-treated H_2_O. RNA was reverse transcribed to cDNA using the Moloney murine leukemia virus reverse transcriptase kit (cat. no. SK021; Evrogen JSC, Moscow, Russia) following the manufacturer’s protocol and further subjected to quantitative PCR. 1 µL of template cDNA in total was used in the qPCR reaction, with 5× qPCRmix-HS SYBR (Evrogen JSC, Moscow, Russia) and 10 mM of each forward and reverse primer (Supplementary Table S1). The sequences of the primers and thermal cycling conditions used in this study are shown elsewhere [18]. qPCR was performed using the CFX96 Real-Time detection system (Bio-Rad Laboratories, Inc., Hercules, CA, USA). Assays for each experience sample were processed in parallel with the reference gene *GAPDH*, and the relative levels of each mRNA were normalized for *GAPDH*. The 2^−∆∆Cq^ method was then used to calculate relative gene expression [19].

### 2.7. Cellular Survival Assay

Parental and SaOS-2_DoxR cells were plated on 96-well flat-bottomed plates (Corning Inc., Corning, NY, USA) and allowed to attach and grow for 24 h before treatment with PTX, Vin, or Dox that were introduced into the cell culture for 72 h with specified concentrations. Half-inhibitory concentrations (IC_50_), further named as IC_50_ values, were defined as the concentration of the anti-cancer drug required to inhibit cellular growth by 50%. This data was normalized to the DMSO-treated control cells. To calculate the IC_50_ values of the chemotherapeutic agents indicated above, MTS reagent (Promega, Madison, WI, USA) was introduced into the cell culture for 1 h to assess the live cell numbers. The viability of the cells was assayed at 492 nm on a MultiScan FC plate reader (Thermo Fisher Scientific, Waltham, MA, USA). IC_50_ values were determined by using the IC_50_ Tool Kit (http://ic50.tk/, accessed on 10 January 2022). 

### 2.8. Real-Time Monitoring of Cell Proliferation

The growth curves of parental and SaOS-2_DoxR cells were analyzed using the iCELLigence system (ACEA Biosciences, San Diego, CA, USA). For this, cells were seeded in electronic microtiter plates (E-Plate; Roche Diagnostics, GmbH, Mannheim, Germany). Cell index measurements were performed with a signal detection set for every 30 min until the end of the experiment (96 h). Normalized cell index values were analyzed by RTCA software, version 1.2 (Roche Diagnostics, GmbH, Mannheim, Germany).

### 2.9. ABC Transporter Assays

The activities of ABC transporters in naive vs. SaOS-2_DoxR cells were assessed by flow cytometry using BD FACSCanto II (Becton Dickinson Biosciences, Franklin Lakes, NJ, USA) and BD FACSDiva Software, version 7.0 following the manufacturer’s protocols. Briefly, cells harvested by trypsinization were pre-incubated for 5 min at room temperature in PBS containing 2% FBS. To examine Calcein AM (ABCB1 and ABCB1-specific probe) and CMFDA (MRP-specific probe)-induced fluorescence, the cells were incubated with the fluorochromes indicated above for 30 min in the dark, further washed twice with PBS, and subjected to Fluorescence-Activated Cell Sorting (FACs) analysis. The specific ABC inhibitors (e.g., cyclosporin, tariquidar, and MK-571) were also used for particular experiments. Concentrations were 10 μM for tariquidar and cyclosporine and 50 μM MK-571. Flow cytometry data were analyzed by comparison of median fluorescence using Kolmogorov–Smirnov statistics (D-value) [20]. 

FACs analysis was also used to examine the efficiency of Dox transport in naive and SaOS-2_DoxR cells. Briefly, cells were trypsinized and incubated with Dox (10 μM) in the dark for 60 min. Subsequently, cells were washed with ice-cold phosphate-buffered saline and analyzed as indicated above. Results were presented as the percentage of desired cells relative to the total number of cells as the mean ± standard deviation (SD) of five biological repeats.

### 2.10. Xenograft Studies Models

Subcutaneous human tumor xenografts were generated via s.c. inoculation in the flank areas of 5-to 8-week-old female nu/nu mice with 100 μL of 1 × 10^7^ SaOS-2 cancer cells/mL suspensions in Dulbecco’s phosphate-buffered saline. The animal experimental protocols were approved by the Committee for Ethics of Animal Experimentation, and the experiments were conducted under the Guidelines for Animal Experiments at the N.N. Blokhin National Medical Research Center of Oncology. Mice were administered three times a week either vehicle (a negative control) or Dox (2.5 mg/kg). The tumor volume, size, and general health of the mice were recorded. After the mice were sacrificed, tumors were excised and subjected to histopathologic examination. Formalin-fixed, paraffin-embedded tissues were sectioned at 4 μM for hematoxylin and eosin (H&E) staining. The images of the IHC-stained samples were captured using an Olympus BX63 microscope (Olympus, Japan).

### 2.11. Statistics 

All the experiments were repeated a minimum of three times. The results are presented as the mean ± standard error (SE) for each group. Statistical analyses (Student’s *t*-test) were performed using Statistical Software Program version 7.0 (S.A. Glantz, McGraw Hill Education, NY, USA). *p* < 0.05 was considered to indicate a statistically significant difference. 

## 3. Results

### 3.1. Establishment of the Multi-Drug Resistant (MDR) SaOS-2 Subline

**Figure 1:**
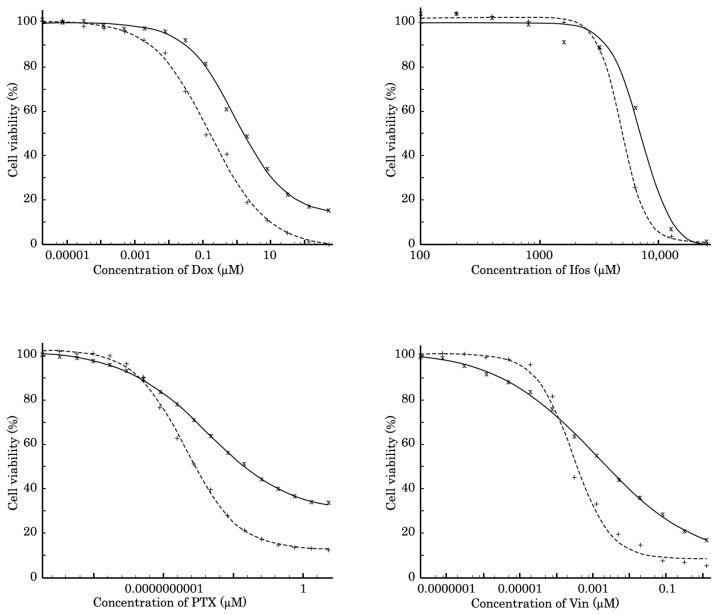
MTS-based viability assay in parental SaOS-2 (dotted line) and SaOS-2_DoxR (solid line) cells treated with Dox, Ifos, PTX, and Vin for 72 h. The data were normalized to DMSO-treated controls.

The SaOS-2 subline resistant to Dox (further named SaOS-2_DoxR) was generated following a continuous treatment of parental SaOS-2 cells with a stepwise increasing concentration of Dox, ranging from 0.01–1 μM. Approximately 5 months and 50 passages were required to develop OS cells with stable drug resistance. The IC_50_ values for the chemotherapeutic agents, including Dox, PTX, Vin, and Ifos, in parental and SaOS-2_DoxR cells are shown in Figure 1 and Table 1. For example, we observed a substantial (~30-fold) increase in the IC_50_ value for PTX in SaOS-2_DoxR cells when compared with naive SaOS-2 cells. Similarly, ~6 and 4-fold increase in IC_50_ values for Dox and Vin was found in SaOS-2_DoxR cells, thereby illustrating the acquisition of the MDR phenotype in this subline. At the same time, we found no significant increase in IC_50_ value for Ifos in SaOS-2_DoxR cells when compared with parental SaOS-2 cells. 

**Figure 2:**
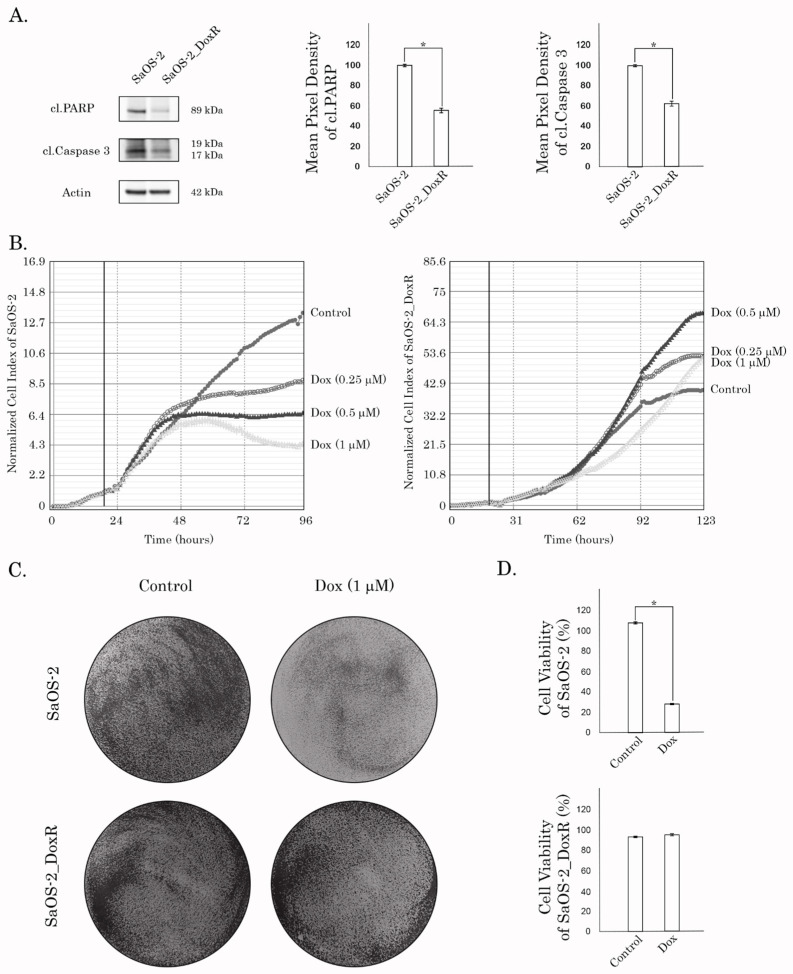
(**A**) Immunoblot analysis for apoptosis markers (i.e., cleaved forms of PARP and caspase-3) in naive and Dox-R SaOS-2 cells after treatment with Dox for 48 h. Actin stain is used as a loading control. Quantification by the mean pixel density of cleaved forms of PARP and caspase-3 in naive and Dox-R SaOS-2 cells as shown in (**A**). Values are means ± SD, *N* = 3. * *p* < 0.05. (**B**) Changes in growth kinetics of SaOS-2 (**left**) and SaOS-2_DoxR (**right**) cells treated with DMSO (control) and Dox (0.25/0.5/1 μM). (**C**) Crystal violet staining of Dox-sensitive and resistant SaOS-2 cells treated with Dox for 72 h. The culture dishes were fixed, stained with crystal violet, and photographed. (**D**) Quantitative analysis of crystal violet staining of SaOS-2 cells as shown in (**C**).

The resistance to Dox in this subline was confirmed by western blotting, which revealed a lack of apoptotic markers (e.g., cleaved PARP and caspase-3) in SaOS-2_DoxR cells treated with this chemotherapeutic agent (Figure 2A). As expected, the expression of these markers was significantly increased in Dox-treated parental SaOS-2 cells (Figure 2A). The resistance to Dox was also observed by the significant decrease in the confluency of parental but not of SaOS-2_DoxR cells after Dox treatment for 72 h (Supplementary Figure S1). Similarly, Dox significantly impaired the proliferative activity of parental but not SaOS-2_DoxR cells (Figure 2B). In addition, crystal violet staining also revealed the decreased viability of Dox-treated naive SaOS-2 cells when compared with SaOS-2_DoxR cells (Figure 2C,D). Resistance to Dox in SaOS-2_DoxR cells remained stable following 1 month of culturing without Dox and following storage at −80 °C for 6 months.

### 3.2. An Increased Expression of ABC-Transporters in the SaOS-2_DoxR Subline Mediates the Effective Efflux of Anti-Cancer Agents

**Figure 3:**
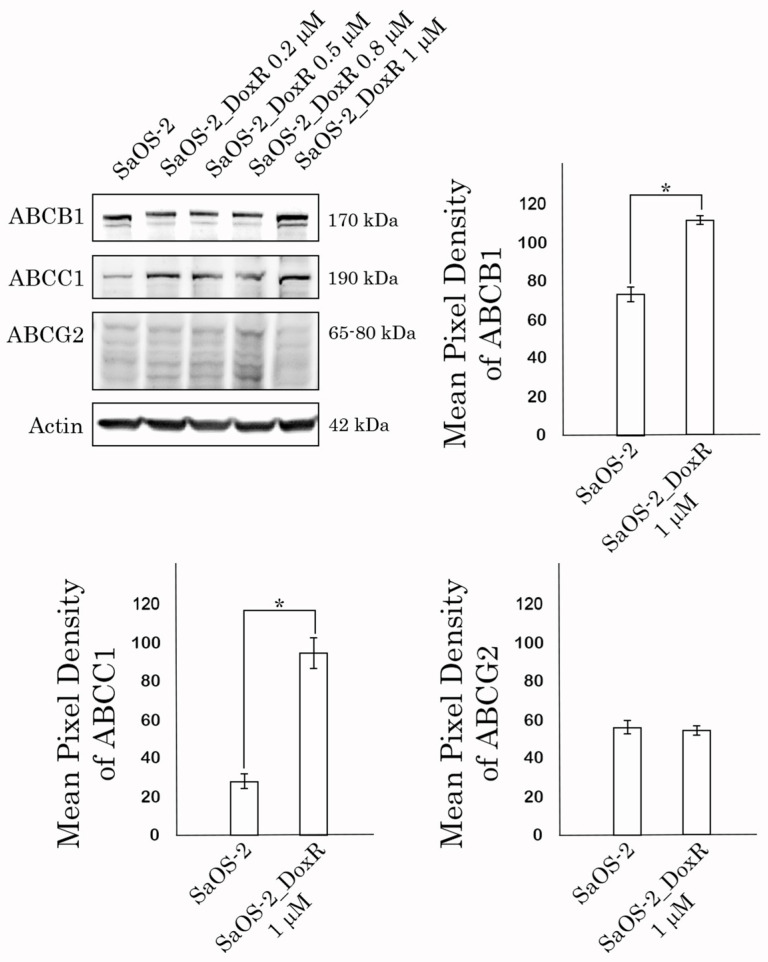
Expression of ABCB1 (P-glycoprotein), ABCC1 (MRP-1), and ABCG2 (BCRP) transporters in naive vs. Dox-resistant SaOS-2 cells. Actin stain was used as a loading control. Quantification by the mean pixel density of ABCB1 (P-glycoprotein), ABCC1 (MRP-1), and ABCG2 (BCRP) transporters in naive vs. Dox-resistant (1 μM) SaOS-2 cells. Values are means ± SD, *N* = 3. * *p* < 0.05.

Given that SaOS-2_DoxR cells exhibited a cross-resistance to certain chemotherapeutic agents indicated above, we initially examined the expression of ABC-transporters providing the efflux of the drugs from cancer cells. Indeed, we found an increased expression of 2 major ABC-transporters—ABCB1 (ATP Binding Cassette subfamily B member 1, also known as P-glycoprotein) and ABCC1 (i.e., multidrug resistance protein-1, MRP-1). In contrast, we observed a minor increase in the expression of ABCG2 (ATP-binding cassette subfamily G member 2, also known as breast cancer resistance protein, BCRP) in the Dox-R subline when compared with the parental SaOS-2 cells. This was observed at both mRNA and protein levels (Table 2 and Figure 3, respectively). Importantly, we observed a time- and dose-dependent increase of MDR1, MRP-1, and ABCG2 expression in SaOS-2 cells treated with Dox (Figure 3). In addition, expression of the other MDR-associated genes (e.g., MRP-4, 5, and -7) was moderately increased in DoxR cells when compared with their parental counterparts (Table 2). Thus, this data demonstrated an increased expression of two major ABC-transporters in the SaOS-2_DoxR subline, thereby suggesting that the MDR phenotype in these cells might be due to the effective efflux of chemotherapeutic agents from cancer cells. 

**Figure 4:**
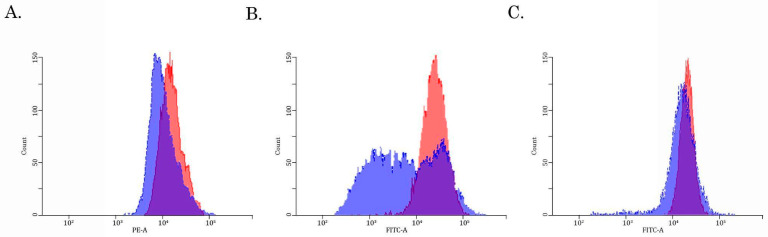
The intracellular accumulation of Dox (**A**), calcein AM (**B**), and CMFDA (**C**) in Dox-resistant (blue) and naive (i.e., drug-sensitive) (red) SaOS-2 cells. The cells were treated with 10 μM of Dox, 100 nM of Calcein AM, or 100 nM CMFDA. The fluorescence intensity was analyzed by FACs. The right-shifting of the latter compared to the former histogram represents inhibition of efflux through the corresponding ABC transporter (representative of 3 samples per group).

To examine this possibility directly, we performed a fluorescence-based assay to compare the Dox-induced intracellular fluorescence between parental and SaOS-2_DoxR cells. For this purpose, naïve vs. resistant SaOS-2 cells were exposed to Dox for 1 h and after the anti-cancer agent was washed out, the cells were subjected to FAC analysis to examine the fluorescence intensity of Dox. FACs data shown in Figure 4A revealed a decreased intensity of Dox fluorescence in SaOS-2_DoxR cells when compared with parental OS cells, thereby revealing an increased efflux of this chemotherapeutic agent from resistant cells.

### 3.3. Assessment of the Activity of the ABCB1-, ABCC1- and ABCG2- Transporters in SaOS-2/Dox-R Cells

**Figure 5:**
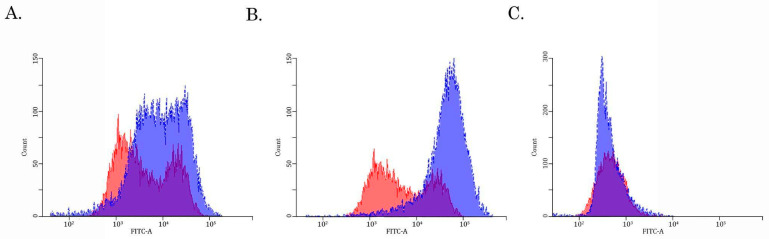
The intracellular accumulation of Calcein AM (**A**,**B**) and CMFDA (**C**) in drug-resistant SaOS-2 cells cultured in the presence of ABC-inhibitors—tariquidar (10 μM) (**A**), cyclosporine (10 μM) (**B**), and MK-571 (50 μM) (**C**). The cells were pretreated with the ABC inhibitors indicated above for 1 h before being exposed to the fluorescent substrates (e.g., Calcein AM and CMFDA) for 30 min. The cells were further washed out with PBS, and fluorescence intensity was analyzed by FACs. Histograms of untreated cells are shown in red, while dark blue indicates histograms obtained after treatment with tariquidar, cyclosporin, or MK-571. The right-shifting of the latter compared to the former histogram represents inhibition of efflux through the corresponding ABC transporter (representative of three samples per group).

Given that expression of ABCB1 and ABCG2 was increased in SaOS-2_DoxR cells, we further examined the functional activities of these ABC-transporters by using the corresponding MDR probes with known substrate specificity (e.g., the Calcein AM (MDR1-specific probe) and the CMFDA (MRP-specific probe). We found a substantial decrease in Calcein AM-mediated fluorescence in Dox-R SaOS-2 cells when compared with parental cancer cells (Figure 4B). In contrast, the fluorescence intensity of CMFDA was similar between parental and SaOS-2_DoxR cells (Figure 4C), thereby suggesting increased activity of the ABCB1 transporter as a prominent mechanism of the efflux of chemotherapeutic agents from SaOS-2_DoxR cells. In addition, a significant fluorescence increase of Calcein AM was observed in the presence of cyclosporine A and tariquidar (potent ABCB1 inhibitors) (Figure 5A,B). As expected, MK-571 has no effect on CMFDA-mediated fluorescence in SaOS-2-DoxR cells (Figure 5C), thereby revealing the inability of the ABCC1 transporter to maintain the efflux of this substrate from SaOS-2 cells. The FSC and SSC characteristics of cells shown in Figure 4 and Figure 5 are shown in Supplementary Figure S2. 

### 3.4. The Activity of DDR Pathways in SaOS-2_DoxR Cell Subline

**Figure 6:**
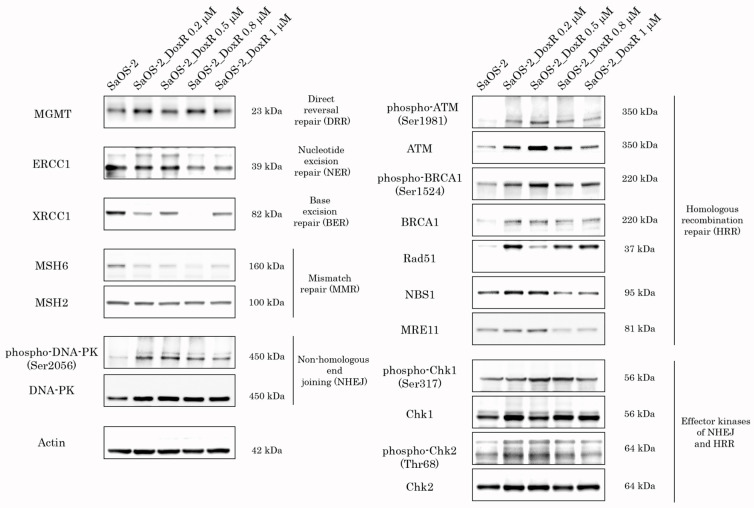
Expression of DDR proteins in SaOS-2 cells during continuous treatment with stepwise increasing concentrations of Dox. Parental SaOS-2 cells were used to assess basal levels of expression of DDR proteins. Actin stain was used as a loading control.

Given that activation of DDR pathways plays an important role in the resistance of cancer cells to chemo- and radiotherapies, we examined the expression of DDR proteins in SaOS-2 cells cultured with stepwise increasing concentrations of Dox for over 5 months. As expected, we observed the activation of DDR pathways involved in the repair of DSBs shortly after the initiation of Dox treatment. Indeed, SaOS-2 cells treated with low doses of Dox (e.g., 0.2 μM) exhibited an increased expression of phosphorylated forms of ATM and DNA-PK-kinases, the well-known sensors of DNA damage (Figure 6), further activating the homology-mediated and non-homology end-joining-mediated DNA repair pathways. Similarly, Chk1 and Chk2 effector kinases were also activated in SaOS-2 cells treated with Dox. In addition, we observed an increased expression of Rad51 recombinase and phosphorylated BRCA1 after Dox treatment, thereby revealing the activation of the homology-mediated DNA repair pathway after induction of DSBs. Lastly, expression of Mre11 and Nbs1, the components of the MRN complex, was moderately increased in Dox-treated cells. In contrast, expression of the proteins involved in nucleotide excision repair (NER), base excision repair (BER), and mismatch repair (MMR) pathways remained unchanged or even decreased after Dox treatment (Figure 6). However, further culturing of the SaOS-2 cells with Dox resulted in a moderate decrease in the expression of the activated forms of DDR proteins that were increased during the initial period. This was shown for both of the sensor and effector kinases involved in DSB repair pathways (e.g., ATM, DNA-PK, Chk1, and Chk2). Indeed, the expression of the phosphorylated forms of these kinases gradually declined after the Dox concentration was increased to 0.8 μM. This discrepancy might be explained by a significant increase in expression of the ABC transporters at this particular time-point (shown in Figure 6), thereby suggesting a decreased intensity of DNA damage in cancer cells due to the effective efflux of the chemotherapeutic agent from cancer cells. 

### 3.5. Anti-Tumor Activity of Dox in SaOS-2 Xenografts

**Figure 7:**
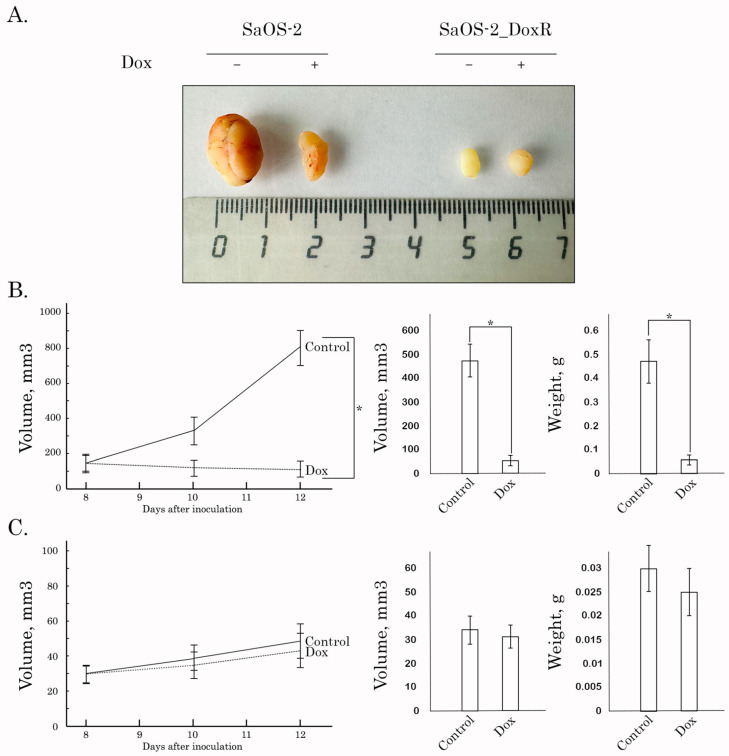
Antitumor effects of Dox in a nude mice xenograft tumor model. After subcutaneous inoculation of naive (**B**) and Dox-R (**C**) SaOS-2 cells (day 8), nude mice were randomized into 2 groups (*n* = 5) and administered i.p. 100 μL of vehicle (negative control) or Dox (2.5 mg/kg). The animals were treated according to the schedule specified in Materials and Methods. The changes in tumor sizes were calculated as a percentage of the baseline. The tumor volume in each group was assessed by calipers and calculated as length × width × width × 0.5. Results were expressed as the mean volume and weight of tumors (mean ± SE, *n* = 5; *: *p* < 0.001) compared to control (vehicle-treated) animals. (**A**) Representative images of the final tumor volumes in each experimental group.

**Figure 8:**
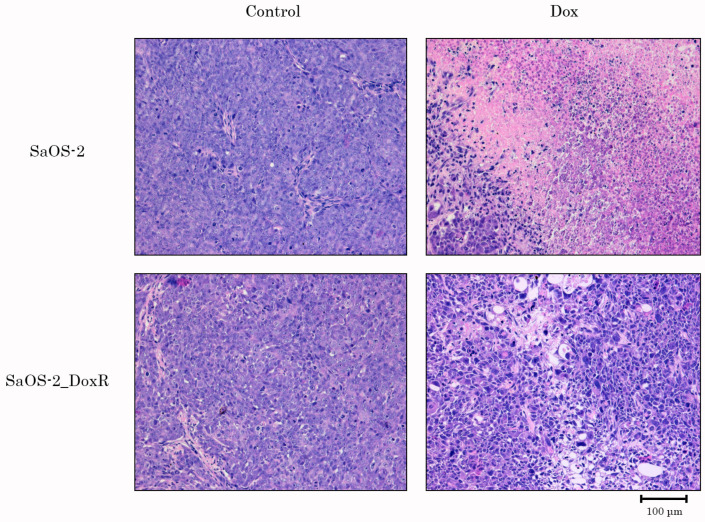
H&E-stained sections of tumors resected from OS xenograft mouse models treated with vehicle (Control) or Dox. Upper line—naive SaOS-2 xenografts; bottom line—SaOS-2_DoxR xenografts. Scale bars: 100 μm.

Lastly, we examined Dox for its anti-tumor activity by using the SaOS-2 vs. SaOS-2_DoxR xenograft models. Of note, the speed of growth of naive tumors was significantly higher when compared with their Dox-R counterparts, as shown in Figure 7A. This might be due to several reasons described in the Discussion. As expected, vehicle-treated mice (control) demonstrated a continuous increase in tumor size from the baseline during the period of the experiment in the next 1 week (Figure 7). A substantial (>70%) decrease in tumor size and volume was observed in Dox-treated mice bearing SaOS-2 tumors. In contrast, SaOS-2-DoxR xenografts exhibited a minor decrease in size and volume after Dox treatment, thereby revealing the resistance of the SaOS-2-DoxR subline to Dox treatment in vivo (Figure 7B,C, respectively). Representative images illustrating changes in tumor sizes in naive and Dox-resistant SaOS-2 tumors after Dox treatment are shown in Figure 7A. H&E staining of the tumors was also performed, which illustrated that both naive and Dox-resistant non-treated tumors comprised dense, viable cancer cells with many mitoses (20–25 mitotic cells per field). Naive SaOS-2 tumors treated with Dox exhibited a much lower density of viable and mitotic cancer cells when compared with control (i.e., non-treated) mice. Necrosis was also found in naive tumors treated with Dox (Figure 8). In contrast, tumors arising from inoculation of SaOS-DoxR cells did not exhibit significant histological changes after Dox treatment compared with vehicle-treated tumors, in addition to a ~2-fold decrease in mitotic cell numbers (Figure 8). 

Collectively, we generated and characterized osteosarcoma SaOS-2 sublines exhibiting stable cross-resistance to a broad spectrum of chemotherapeutic agents (e.g., Dox, Vin, PTX) due to the increased activity of ABC-transporters and activation of DDR pathways involved in DSB DNA repair during the initial steps of the acquisition of drug resistance. 

## 4. Discussion

The acquisition of resistance to chemotherapeutic agents in cancer patients remains a major obstacle in anti-cancer treatment, decreasing progression-free survival and overall survival as well. Malignant tumors acquire the multi-drug resistance (MDR) phenotype via a broad spectrum of overlapping molecular mechanisms, especially at the final stage of the disease. This includes an increased efflux of anti-cancer drugs, activation of DNA repair pathways, attenuated apoptosis, epithelial-mesenchymal transition, etc. (reviewed in [21,22,23] or elsewhere). In particular, increased Pgp protein and *MDR1* gene expression in tumors correlate with resistance to chemotherapeutic agents [24]. Indeed, the expression of Pgp in cancer cells has often been associated with poor prognosis and failure of chemotherapy [25]. Therefore, the elucidation of the underlying pleiotropic mechanisms involved in the resistance of cancer cells to chemotherapeutic drugs used for the therapy of different types of malignancies is essential for the development of novel strategies to effectively overcome the resistance mechanisms in tumors [26,27]. Furthermore, non-target-specific chemotherapeutic agents usually exhibit low bioavailability and are generally toxic, and their efficiency is limited by multiple side effects, including cardiotoxicity, myelo- and immunosuppression, neutropenia, mucositis, and fluid retention. Thus, there is an urgent need for the development of novel, safe, and effective therapeutic options for cancer therapies to bypass MDR mechanisms. To achieve this goal, multiple strategies aimed at reversing the MDR phenotype of cancer cells have been extensively studied over the past few decades. For example, 3 generations of MDR inhibitors have been developed, including verapamil, valspodar (PSC833), biricodar (VX710), tariquidar (XR9576), zosuquidar (LY335979), laniquidar (R101933), etc. [28,29]. Several clinical trials of the MDR inhibitors indicated above have been conducted in various cancer types [30,31,32,33]. Unfortunately, almost no substantial survival benefits have been established, which might be due to the pleiotropic molecular mechanisms of drug resistance ongoing at the single cancer cell level. 

Our data shown here illustrates the acquisition of the MDR phenotype in a p53-negative SaOS-2 cell line cultured in the presence of stepwise increasing concentrations of Dox for 5 months. Resistance to chemotherapeutic agents, including Dox, Vin, and PTX, was developed due to pleiotropic molecular mechanisms, including an increased efflux of the anti-cancer agents and DDR mechanisms involved in the repair of DSBs. 

The first one was due to the up-regulation of several ABC-transporters (e.g., P-glycoprotein and MRP-1), which was evidenced both on the mRNA and protein levels (Table 1, Figure 3). Increased activity of the ABC-transporters was revealed by the decreased intracellular concentration of Dox in SaOS-2_DoxR cells when compared with parental SaOS-2 cells (Figure 4A). Since Dox is a well-known substrate for ABCB1 (P-glycoprotein) and ABCC1 (MRP-1) transporters [34,35], the decreased fluorescence intensity of this fluorescent drug in SaOS-2_DoxR cells indicates its increased efflux from cancer cells via the ABC-transporters indicated above. To assess the functional activity of ABC-transporters more precisely in SaOS-2_DoxR cells, we utilized several ABC transporter substrates to evaluate their intracellular accumulation. In particular, Calcein AM is known to be a substrate for P-glycoprotein and MRP-1 [36,37]. It easily gets into the cells via passive diffusion and becomes cleaved irreversibly to a hydrophilic, non-permeable, and fluorescent calcein AM-free acid by endogenous esterases. Therefore, intracellular accumulation of the fluorescent calcein AM is inversely related to ABC-mediated efflux from the cells. Indeed, we observed a decreased intensity of calcein AM-mediated fluorescence in SaOS-2_DoxR, as shown in Figure 4B. This might indicate increased activity of ABCB1 and ABCC1 transporters in the MDR SaOS-2 subline. In contrast to Calcein AM, FACs data with CMFDA, a well-known specific substrate for ABCC1 [38], demonstrated similar intensity of fluorescence in naive and Dox-resistant SaOS-2 cells (Figure 4C), thereby indicating the efflux of Dox and Calcein AM from SaOS-2_DoxR was mainly mediated via the ABCB1 transporter. 

Of note, we also observed the activation of DDR pathways involved in DSB DNA repair in SaOS-2 cells exposed to Dox. As expected, this happened in OS cells at the early beginning of treatment with Dox (Figure 6). However, the signs of activation of DDR proteins during long-term exposure to Dox eventually decreased. Of note, decreased expression of the activated forms of ATM, DNA-PK, BRCA1, and Chk1/2 kinases (Figure 6) is inversely related to an increased expression of ABC-transporters (Figure 3), thereby illustrating the decreased intensity of DNA damage in Dox-treated OS cells due to an increased efflux of the chemotherapeutic agents (e.g., Dox) from cancer cells. 

Importantly, resistance to Dox in SaOS-2_DoxR cells was also illustrated by the in vivo studies using a xenograft model (Figure 7 and Figure 8). Surprisingly, 8 days after subcutaneous inoculation of cancer cells, SaOS-2_DoxR tumors exhibited much lower sizes and volumes when compared with naive SaOS-2 tumors. This might be due to several reasons, including the higher metastatic potential of SaOS-2_DoxR cells and/or the enhanced mutational burden of resistant tumor cells, thereby triggering a potent immune response and reducing the size and volume of the primary tumor. Additional experiments (comparative analysis of migration and invasion capacities in naive vs. resistant OS cells) and histological examination of the primary tumors and places of potential metastatic lesions (e.g., regional lymphatic nodes, lungs, and liver) are needed to confirm this hypothesis. Despite the fact that SaOS-2_DoxR-based xenografts exhibited lower volumes and sizes when compared with naive SaOS-2 tumors, they demonstrated resistance to Dox treatment. This was in agreement with our in vitro studies shown before. 

Collectively, we established and characterized the SaOS-2_DoxR subline as exhibiting cross-resistance to several chemotherapeutic agents, including Dox, Vin, and PTX. This multi-drug-resistant OS cell subline can be suitable for understanding the multifactorial resistance processes in tumor cells and, consequently, for screening novel anti-cancer drugs to overcome the mechanisms of resistance found in the clinical context.

## Figures and Tables

**Table 1:** The IC_50_ values for Dox, Ifos, PTX, and Vin in parental SaOS-2 and SaOS-2_DoxR cells.

Chemical Compounds	Parental	Dox-R	Fold Increase
Dox (μM)	0.16 ± 0.02	0.97 ± 0.12	6.1
Ifos (μM)	4903 ± 91	7197 ± 618	1.5
PTX (μM)	0.0000000039 ± 0.0000000008	0.0000001196 ± 0.0000000209	30.7
Vin (μM)	0.00029 ± 0.00005	0.00122 ± 0.00021	4.2

The data were normalized to DMSO-treated controls. Values are the means ± SD (*n* = 3).

**Table 2:** Fold-increase of ABC-transporter mRNA levels in SaOS-2_DoxR cells compared with parental SaOS-2 cells (determined by RT-qPCR).

Genes	*MDR-1*	*MRP-1*	*MRP-2*	*MRP-3*	*MRP-4*	*MRP-5*	*MRP-7*	*ABCG2*
Fold-increase	20.2	19.6	0.02	1.1	2.1	2.0	4.0	1.2

Values are averages of three experiments.

## Data Availability

The data and materials shown in the present manuscript are available from the corresponding authors upon reasonable request.

## Supplementary Materials

The following supporting information can be downloaded at: https://www.mdpi.com/article/10.3390/diagnostics13162646/s1, Figure S1: Dox induces the changes in the confluency of SaOS-2 cells. SaOS-2 and SaOS-2_DoxR cells were treated with solvent (DMSO) (control) and Dox (1 μM) for 72 h and subjected to the light microscopy (Leica, 10×); Figure S2: FSC and SSC characteristics of Dox-resistant and naive (i.e., drug-sensitive) SaOS-2 cells that were shown in Figure 4A or Figure 5B; Table S1: Primers for quantitative polymerase chain reaction.

## Abbreviations

Osteosarcoma	OS
doxorubicin	Dox
half-inhibitory concentrations	IC_50_
vinblastine	Vin
paclitaxel	PTX
the multidrug resistance	MDR
DNA damage response	DDR
Dox-resistant OS subline	SaOS-2_DoxR
double-strand breaks	DSBs
Ifosfamide	Ifos
Calcein acetoxymethyl ester	Calcein AM
dimethyl sulfoxide	DMSO
Fluorescence-Activated Cell Sorting	FACs
standard deviation	SD
hematoxylin and eosin	H&E
standard error	SE
ATP Binding Cassette subfamily B member 1	ABCB1, also known as P-glycoprotein
ATP Binding Cassette subfamily C member 1	ABCC1, also known as multidrug resistance protein-1, MRP-1
ATP-binding cassette subfamily G member 2	ABCG2, also known as breast cancer resistance protein, BCRP

## References

[1] Rutkowski P., Świtaj T. (2018). Bone sarcomas. Oncol. Clin. Pract..

[2] Wang H., Miao R., Jacobson A., Goldberg S., Harmon D.C., Cote G., Hornicek F.J., Raskin K., Nielsen G., DeLaney T.F. (2017). Osteosarcoma prognostic nomograms for predicting the 10-year probability of mortality and recurrence. J. Clin. Oncol..

[3] Trock B.J., Leonessa F., Clarke R. (1997). Multidrug resistance in breast cancer: A meta-analysis of MDR1/gp170 expression and its possible functional significance. J. Natl. Cancer Inst..

[4] Burger H., Foekens J.A., Look M.P., Meijer-van Gelder M.E., Klijn J.G., Wiemer E.A., Stoter G., Nooter K. (2003). RNA expression of breast cancer resistance protein, lung resistance-related protein, multidrug resistance-associated proteins 1 and 2, and multidrug resistance gene 1 in breast cancer: Correlation with chemotherapeutic response. Clin. Cancer Res..

[5] Lord C.J., Ashworth A. (2013). Mechanisms of resistance to therapies targeting BRCA mutant cancers. Nat. Med..

[6] Viktorsson K., Lewensohn R., Zhivotovsky B. (2005). Apoptotic pathways and therapy resistance in human malignancies. Adv. Cancer Res..

[7] Hersey P., Zhang X.D., Mhaidat N. (2008). Overcoming resistance to apoptosis in cancer therapy. Adv. Exp. Med. Biol..

[8] Borst P. (2012). Cancer drug pan-resistance: Pumps, cancer stem cells, quiescence, epithelial to mesenchymal transition, blocked cell death pathways, persisters or what?. Open Biol..

[9] Zheng X., Carstens J.L., Kim J., Scheible M., Kaye J., Sugimoto H., Wu C.C., LeBleu V.S., Kalluri R. (2015). Epithelial-to-mesenchymal transition is dispensable for metastasis but induces chemoresistance in pancreatic cancer. Nature.

[10] Du B., Shim J.S. (2016). Targeting Epithelial-Mesenchymal Transition (EMT) to overcome drug resistance in Cancer. Molecules.

[11] Fischer K.R., Durrans A., Lee S., Sheng J., Li F., Wong S.T., Choi H., El Rayes T., Ryu S., Troeger J. (2015). Epithelial-to-mesenchymal transition is not required for lung metastasis but contributes to chemoresistance. Nature.

[12] Lorenz S., Barøy T., Sun J., Sun J., Nome T., Vodák D., Bryne J.C., Håkelien A.M., Fernandez-Cuesta L., Möhlendick B. (2016). Unscrambling the genomic chaos of osteosarcoma reveals extensive transcript fusion, recurrent rearrangements and frequent novel TP53 aberrations. Oncotarget.

[13] Chen X., Bahrami A., Pappo A., Easton J., Dalton J., Hedlund E., Ellison D., Shurtleff S., Wu G., Wei L. (2014). Recurrent somatic structural variations contribute to tumorigenesis in pediatric osteosarcoma. Cell. Rep..

[14] Mirabello L., Zhu B., Koster R., Karlins E., Dean M., Yeager M., Gianferante M., Spector L.G., Morton L.M., Karyadi D. (2020). Frequency of Pathogenic Germline Variants in Cancer-Susceptibility Genes in Patients With Osteosarcoma. JAMA Oncol..

[15] Chen Z., Guo J., Zhang K., Guo Y. (2016). TP53 Mutations and Survival in Osteosarcoma Patients: A Meta-Analysis of Published Data. Dis. Markers.

[16] Selvarajah J., Nathawat K., Moumen A., Ashcroft M., Carroll V.A. (2013). Chemotherapy-mediated p53-dependent DNA damage response in clear cell renal cell carcinoma: Role of the mTORC1/2 and hypoxia-inducible factor pathways. Cell Death Dis..

[17] Ye S., Shen J., Choy E., Yang C., Mankin H., Hornicek F., Duan Z. (2016). p53 overexpression increases chemosensitivity in multidrug-resistant osteosarcoma cell lines. Cancer Chemother Pharmacol..

[18] Boichuk S., Galembikova A., Sitenkov A., Khusnutdinov R., Dunaev P., Valeeva E., Usolova N. (2017). Establishment and Characterization of a Triple Negative Basal-like Breast Cancer Cell Line with Multi-Drug Resistance. Oncol. Lett..

[19] Kuang Y.-H., Shen T., Chen X., Sodani K., Hopper-Borge E., Tiwari A.K., Lee J.W.K.K., Fu L.-W., Chen Z.-S. (2010). Lapatinib and Erlotinib Are Potent Reversal Agents for MRP7 (ABCC10)-Mediated Multidrug Resistance. Biochem. Pharmacol..

[20] Young I.T. (1977). Proof without prejudice: Use of the Kolmogorov-Smirnov test for the analysis of histograms from flow systems and other sources. J. Histochem. Cytochem..

[21] Aleksakhina S.N., Kashyap A., Imyanitov E.N. (2019). Mechanisms of Acquired Tumor Drug Resistance. Biochim. Biophys. Acta Rev. Cancer.

[22] Kachalaki S., Ebrahimi M., Khosroshahi L.M., Mohammadinejad S., Baradaran B. (2016). Cancer chemoresistance; biochemical and molecular aspects: A brief overview. Eur. J. Pharm. Sci..

[23] Salehan M.R., Morse H.R. (2013). DNA damage repair and tolerance: A role in chemotherapeutic drug resistance. Br. J. Biomed. Sci..

[24] Triller N., Korosec P., Kern I., Kosnik M., Debeljak A. (2006). Multidrug resistance in small cell lung cancer: Expression of P-glycoprotein, multidrug resistance protein 1 and lung resistance protein in chemo-naive patients and in relapsed disease. Lung Cancer.

[25] Gottesman M.M., Fojo T., Bates S.E. (2002). Multidrug resistance in cancer: Role of ATP-dependent transporters. Nat. Rev. Cancer.

[26] Zahreddine H., Borden K. (2013). Mechanisms and insights into drug resistance in cancer. Front. Pharm..

[27] Wang J., Seebacher N., Shi H., Kan Q., Duan Z. (2017). Novel strategies to prevent the development of multidrug resistance (MDR) in cancer. Oncotarget.

[28] Palmeira A., Sousa E., Vasconcelos M.H., Pinto M.M. (2012). Three decades of P-gp inhibitors: Skimming through several generations and scaffolds. Curr. Med. Chem..

[29] Lai J.I., Tseng Y.J., Chen M.H., Huang C.F., Chang P.M. (2020). Clinical Perspective of FDA Approved Drugs With P-Glycoprotein Inhibition Activities for Potential Cancer Therapeutics. Front. Oncol..

[30] Baekelandt M., Lehne G., Trope C.G., Szanto I., Pfeiffer P., Gustavssson B., Kristensen G.B. (2001). Phase I/II trial of the multidrug-resistance modulator valspodar combined with cisplatin and doxorubicin in refractory ovarian cancer. J. Clin. Oncol..

[31] Fracasso P.M., Brady M.F., Moore D.H., Walker J.L., Rose P.G., Letvak L., Grogan T.M., McGuire W.P. (2001). Phase II study of paclitaxel and valspodar (PSC 833) in refractory ovarian carcinoma: A gynecologic oncology group study. J. Clin. Oncol..

[32] Seiden M.V., Swenerton K.D., Matulonis U., Campos S., Rose P., Batist G., Ette E., Garg V., Fuller A., Harding M.W. (2002). A phase II study of the MDR inhibitor biricodar (INCEL, VX-710) and paclitaxel in women with advanced ovarian cancer refractory to paclitaxel therapy. Gynecol. Oncol..

[33] Lhomme C., Joly F., Walker J.L., Lissoni A.A., Nicoletto M.O., Manikhas G.M., Baekelandt M.M., Gordon A.N., Fracasso P.M., Mietlowski W.L. (2008). Phase III study of valspodar (PSC 833) combined with paclitaxel and carboplatin compared with paclitaxel and carboplatin alone in patients with stage IV or suboptimally debulked stage III epithelial ovarian cancer or primary peritoneal cancer. J. Clin. Oncol..

[34] Bosch I., Croop J. (1996). P-glycoprotein multidrug resistance and cancer. Biochim. Biophys. Acta.

[35] Sampson A., Peterson B.G., Tan K.W., Iram S.H. (2019). Doxorubicin as a fluorescent reporter identifies novel MRP1 (ABCC1) inhibitors missed by calcein-based high content screening of anticancer agents. Biomed. Pharmacother..

[36] Hollo Z., Homolya L., Davis C.W., Sarkadi B. (1994). Calcein accumulation as a fluorometric functional assay of the multidrug transporter. Biochim. Biophys. Acta.

[37] Lebedeva I.V., Pande P., Patton W.F. (2011). Sensitive and specific fluorescent probes for functional analysis of the three major types of mammalian ABC transporters. PLoS ONE.

[38] Roelofsen H., Vos T.A., Schippers I.J., Kuipers F., Koning H., Moshage H., Jansen P.L., Müller M. (1997). Increased levels of the multidrug resistance protein in lateral membranes of proliferating hepatocyte-derived cells. Gastroenterology.

