# Supplementary material for: Establishment and Characterization of Multi-Drug Resistant p53-Negative Osteosarcoma SaOS-2 Subline
Source: Diagnostics (Basel). 2023 Aug 11;13(16):2646. doi: 10.3390/diagnostics13162646 (PMC10453552; doi:10.3390/diagnostics13162646)
Supplement: Supplementary file 1 [file diagnostics-13-02646-s001.zip › diagnostics-2532880-supplementary.pdf]

Figure legends

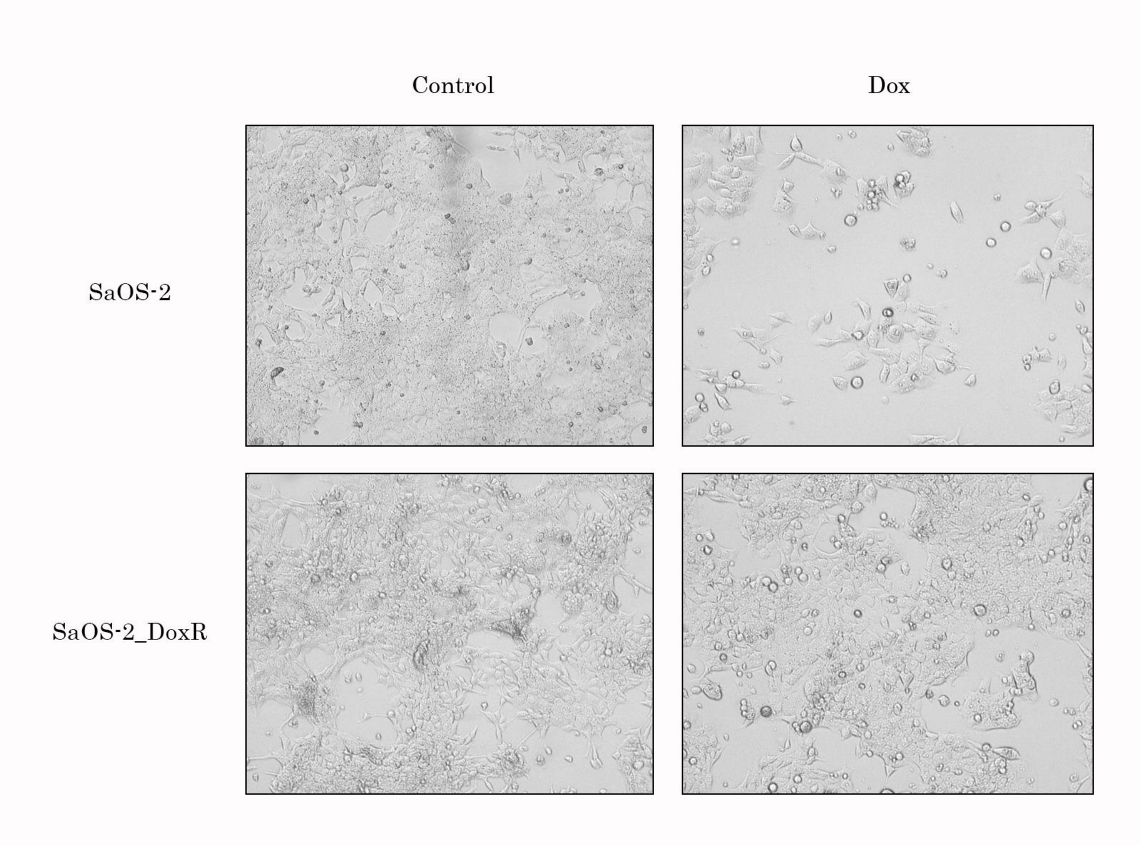

Supplementary Figure S1. Dox induces the changes in the confluency of SaOS-2 cells. SaOS-2 and SaOS-2_DoxR cells were treated with solvent (DMSO) (control) and Dox (1 μM) for 72 h and subjected to the light microscopy (Leica, 10×).

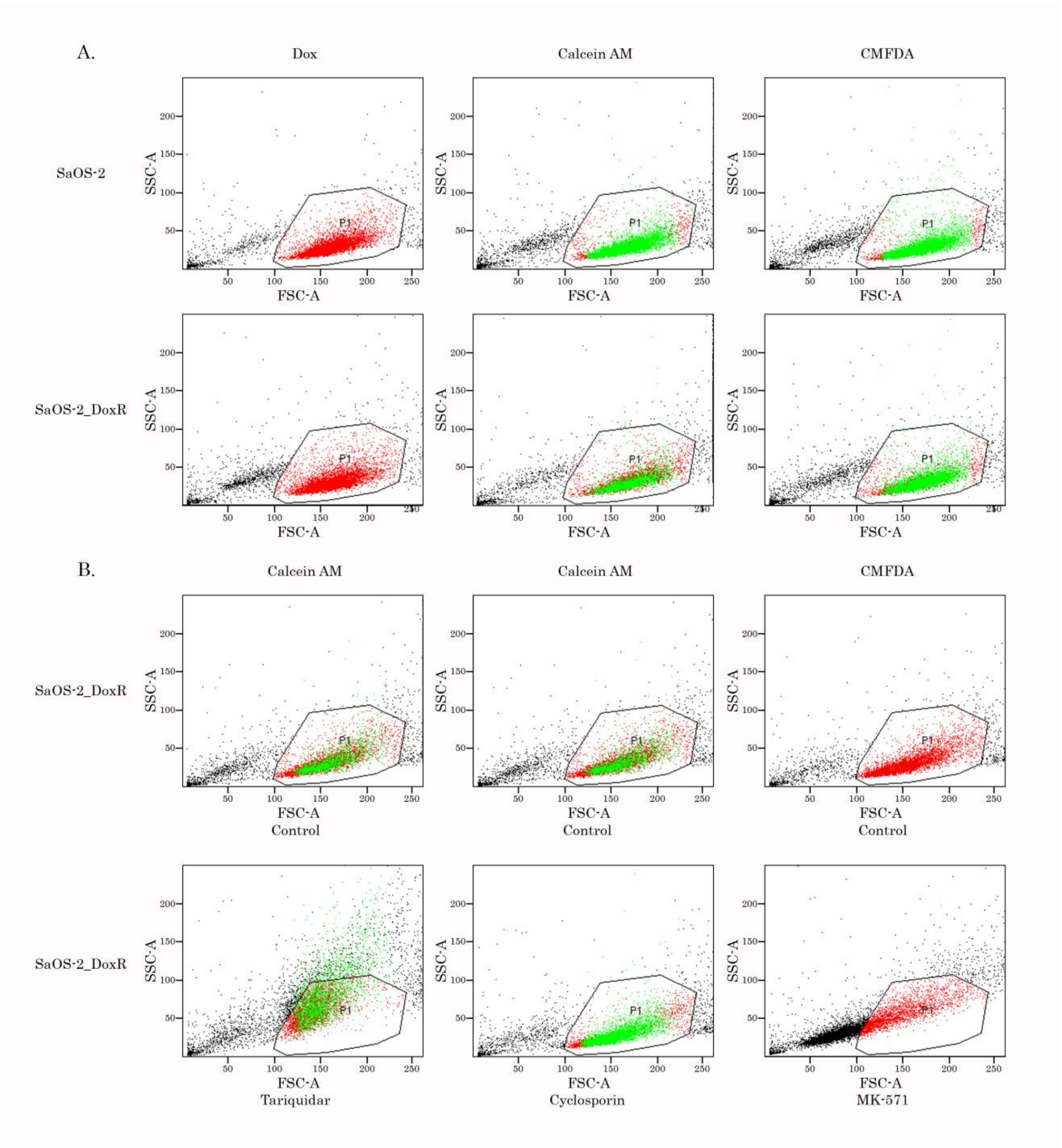

Supplementary Figure S2. FSC and SSC characteristics of Dox-resistant and naive (i.e., drug-sensitive) SaOS-2 cells that were shown in Figure 4 (A) or Figure 5 (B).

Supplementary Table S1. Primers for quantitative polymerase chain reaction

Gene	Forward	Reverse
GAPDH	GACCACAGTCCATGCCATCA	TCCACCACCCTGTTGCTGTA
MDR-1	ATGCTCTGGCCTTCTGGATGGGA	ATGGCGATCCTCTGCTTCTGCCCAC
MRP-1	GCATGA TCCCTGAAGACGA	TAGAGCTGGCCCTTGTACTC
MRP-2	TAGAGCTGGCCCTTGTACTC	TCAACTTCCCAGACATCCTC
MRP-3	CGCCTGTTTTTCTGGTGGTT	TCCCCCAGTCACAAAGATG
MRP-4	GCTGAGAATGACGCACAGAA	TCCCAGCAAGGCACGATATT
MRP-5	GTCCTGGGTATAGAAGTGTG	CAGAAGATCCACACAACCCT
MRP-6	TTGGATTCGCCCTCATAGTC	TCTTTTGGTCTCAGTGGCCT
MRP-7	CTCCCACTGGATCTCTCAGC	TCGCATACACGGTGAGGTAG
ABCG2	TTTCCAAGCGTTCATTCAAAA A	TACGACTGTGACAATGATCTGAGC
